# Twin-mediated crystal growth: an enigma resolved

**DOI:** 10.1038/srep28651

**Published:** 2016-06-27

**Authors:** Ashwin J. Shahani, E. Begum Gulsoy, Stefan O. Poulsen, Xianghui Xiao, Peter W. Voorhees

**Affiliations:** 1 Department of Materials Science and Engineering, Northwestern University, Evanston, IL 60208, USA; 2 Advanced Photon Source, Argonne National Laboratory, Lemont, IL 60439, USA

## Abstract

During crystal growth, faceted interfaces may be perturbed by defects, leading to a rich variety of polycrystalline growth forms. One such defect is the coherent Σ3 {111} twin boundary, which is widely known to catalyze crystal growth. These defects have a profound effect on the properties of many materials: for example, electron-hole recombination rates strongly depend on the character of the twin boundaries in polycrystalline Si photovoltaic cells. However, the morphology of the twinned interface during growth has long been a mystery due to the lack of four-dimensional (i.e., space and time resolved) experiments. Many controversial mechanisms have been proposed for this process, most of which lack experimental verification. Here, we probe the real-time interfacial dynamics of polycrystalline Si particles growing from an Al-Si-Cu liquid *via* synchrotron-based X-ray tomography. Our novel analysis of the time evolution of the interfacial normals allows us to quantify unambiguously the habit plane and grain boundary orientations during growth. This, when combined with direct measurements of the interfacial morphology provide the first confirmation of twin-mediated growth, proposed over 50 years ago. Using the insights provided by these experiments, we have developed a unified picture of the phenomena responsible for the dynamics of faceted Si growth.

Polycrystalline materials play a central role in everyday life, ranging from medical devices to airplane wings. The physical properties of the polycrystalline solid depend on the three-dimensional network of internal interfaces, i.e., the grain boundaries. In particular, polycrystalline Si (poly-Si) is widely used as the substrate of thin-film photovoltaic (PV) cells. To be commercially relevant, the efficiency of the poly-Si thin-film cells should reach 12% offered by other thin-film PV technologies^1^. However, the highest recorded efficiencies of the poly-Si thin-film cells are approx. 8–10%^2^,^3^, due to the high defect densities in the poly-Si thin films. Recombination at dislocations and grain boundaries adversely impact the performance of these devices^1^. For instance, the minority carrier lifetime depends on the character of the grain boundaries: coherent Σ3 (twin) boundaries are electrically inactive, while higher-order boundaries may be electrically active^4^,^5^. To clarify the nomenclature, in coincident site lattice (CSL) theory, Σ*m* describes the “degree of fit” between two adjacent grains; the positive integer *m* is the reciprocal of the ratio of the coinciding sites to the total number of sites^6^. Thus, a fundamental understanding of the structure of polycrystalline materials during growth will provide the strategic link between solidification microstructure and underlying materials behavior.

When group *IV* semiconductor crystals such as Si and Ge (point group *m*3*m*) precipitate from the melt, they form faceted crystals with {111} habit planes. {111} is the densely packed cleavage plane in the diamond cubic structure; it is now well established that {111} is both the low mobility^7^,^8^ and low energy^9^,^10^ orientation in such materials. The growth of faceted crystals requires large supersaturations in the melt unless there is some defect along the solid-liquid interface that facilitates the formation of new atomic layers, without the necessity of two-dimensional (2-D) nucleation on the {111} habit planes. Frank^11^,^12^ was the first to suggest that the steady-state growth of imperfect crystals is possible if there is a self-perpetuating step on the growth surfaces. These self-perpetuating steps result from two sources: (i) screw dislocations, which promote continuous spiral growth of atomic layers^11^, and (ii) twinned interfaces, which reduce the nucleation barrier by the line energy at the re-entrant groove^13^. Therefore, defective crystals can nucleate and grow rapidly with very little undercooling below the liquidus, compared to single crystals.

The growth model of a twinned diamond cubic crystal was first proposed independently by Wagner, and by Hamilton and Seidensticker in 1960^14^,^15^. We here review this twin-mediated growth mechanism, hereafter referred to as the WHS model. The central assumption of WHS theory is that nucleation of new layers takes place at the concave 141.06° re-entrant grooves that are caused by the intersection of the {111} Σ3 twin planes with the surface. According to simple bonding arguments, an atom adsorbed on the groove has four nearest neighbors, compared to three on a flat {111} plane^16^,^17^, and thus the groove may act as a preferential site for solute adsorption. A singly-twinned crystal contains three such re-entrant grooves along the 〈211〉 directions. Since nucleation readily occurs at the re-entrant grooves compared to the {111} surfaces, the crystal grows rapidly at the re-entrant grooves and a trigonal solid with 60° corners is obtained. Rapid growth is terminated at the time owing to the disappearance of the re-entrant groove, hence why a multiply twinned interface is needed for steady-state crystal growth. In other words, the solid is bounded by convex 218.94° ridge structures; since an adatom has only three nearest neighbors on either side of the ridge^16^, the ridges are not capable of continuous propagation. For a crystal with two parallel twin planes, as shown in Supplementary Fig. 1, rapid growth occurs at the 141.06° re-entrant corner (referred to as a type *I* corner^15^), similar to the crystal with one twin. When the nucleated layer propagates to the next twin, it forms a new re-entrant corner with an angle of 109.47° (referred to as a type *II* corner^15^). Like the type *I* corner site, the type *II* corner site is four-fold coordinated^17^. The type *II* corner is essential for the continuous propagation of the crystal interface because it relieves the shortage of nucleation sites caused by the formation of ridge structures. The important feature of the WHS model is that the type *I* corner does not disappear during growth, since it is regenerated by activity at the type *II* corner^14^,^15^. In addition, several authors^16^,^18^,^19^ suggest alternative growth mechanisms of a twinned interface involving the atomically rough {100} habit plane, but they disagree on the precise role of the {100} surface during microstructural evolution. In general, while it is widely accepted that at least two twin planes are needed for steady-state growth, the growth behavior of the twinned interface remains poorly understood due to the lack of *in situ* experimental evidence.

Recently, Fujiwara and colleagues^20^,^21^,^22^,^23^ developed a 2-D *in situ* observation system to watch the growth of faceted Si dendrites. Based on this data, they proposed a growth mechanism of a doubly-twinned interface, hereby denoted as the F model and depicted in Supplementary Fig. 2. In their approach, triangular 60° corners (at the length-scale of the crystal) form at the growth tip, and the direction of the 60° corners changes during growth; this is made possible by the alternate formation and disappearance of type *I* corners at each of the two twin planes, in contrast to the WHS mechanism. Thus, according to the F model, type *II* corners play no role during growth^20^,^21^. While this model satisfactorily explains the observations of Fujiwara and colleagues, many open questions remain: namely, how do the experimental conditions (e.g., temperature, undercooling, and composition) influence the morphology of the twinned interface? To what extent is the F model applicable to other materials systems and solidification pathways? Furthermore, the studies conducted by Fujiwara and colleagues^20^,^21^,^22^,^23^ are limited to examing 2-D views of the material. While one can extract some qualitative information from these 2-D images, most kinetic models (e.g., solidification^24^, coarsening^25^, etc.) make predictions based upon a 3-D microstructure.

To circumvent the above challenges, we probe the realtime interfacial dynamics during the growth of Si particles in an Al-Si-Cu liquid *via* 4-D (i.e., 3-D space plus time) synchrotron-based X-ray tomography (XRT). Our subsequent analysis of interfacial texture allows us to quantify unambiguously the habit plane and grain boundary orientations during growth, from which we validate with high precision the aforementioned twin-mediated growth mechanisms. To the best of our knowledge, this is the first time that time-dependent crystallographic information has been obtained from attenuation contrast XRT. We believe that this novel experiment and analysis method provide a unique approach for understanding crystal growth, and will have significant impacts on the processing of polycrystalline materials.

## Results

### Macrostructural Characterization

**Figure 1:**
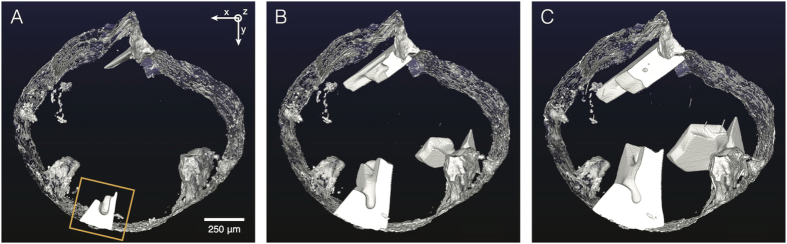
3-D reconstructions showing the growth process of Si particles in a liquid. White regions are the primary Si particles, translucent light gray region is the oxide skin, and the dark blue background is the Cu-enriched liquid. Shown are three representative time-steps during the *in situ* XRT scan: (**A**) 2079 s (875 °C), (**B**) 4261 s (839 °C), and (**C**) 8031 s (776 °C). The time *t* = 0 s (910 °C) corresponds to the onset of the XRT scan, at which the sample is entirely liquid. The field-of-view measures 1664 × 1664 × 715 *μ*m^3^. The specimen coordinate system 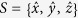 is indicated in (**A**), where 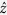 points out of the page and is parallel to the axis of rotation of the cylindrical sample. The boxed particle in (**A**) is considered for further analysis.

The evolution of the sample at three representative time-steps is shown in Fig. 1. White regions correspond to the primary Si particles, and the dark blue background to the Al-Si-Cu liquid. Visible in Fig. 1(B,C) are four Si particles that grow from the oxide skin into the melt. Two of the particles appear to be in contact at the latest time. These reconstructions reflect the extraordinary morphological complexity of the primary Si particles during crystallization. Note that the weight fraction of the Si phase versus temperature in this experiment is almost coincident with predictions at equilibrium (see Supplementary Fig. 3). However, the shapes of the Si particles in Fig. 1 do not resemble the equilibrium Wulff shape of the crystal, a tetrakaidecahedron dominated by {111} habit planes^10^. Instead, defects that perturb the solid-liquid interfaces may lead to a rich variety of polycrystalline growth forms.

**Figure 2:**
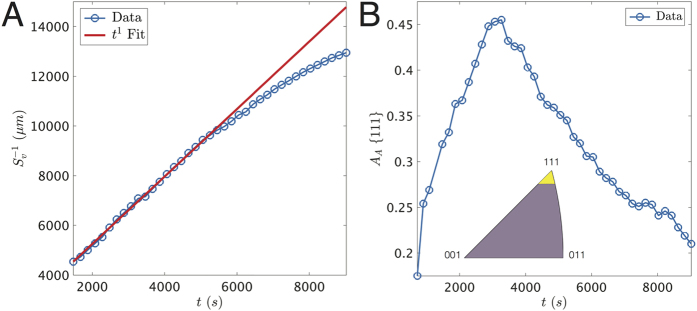
(**A**) Inverse surface area per unit volume 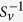 versus time *t* for the boxed particle in Fig. 1(A). To the experimental data, in blue, and in the range 1500 ≤ *t* ≤ 5000 s, we fit a line of constant slope, in red, corresponding to interface-reaction-limited growth (*R*^2^ = 0.99). (**B**) Small patches along the solid-liquid interfaces are indexed as {111} or near-{111} if the orientation of such patches falls within the yellow region of the IPF (see inset). The total area fraction, *A*_*A*_, of these {111} patches relative to the original, parent crystal is plotted over time *t*. *A*_*A*_ {111} passes through a maximum of approx. 0.45 at 3200 s, suggesting that facet growth at early times (*t* < 3200 s) competes with the formation of twinned orientations at later times (*t* > 3200 s).

We consider the boxed particle in Fig. 1 for further analysis. We calculate for this particle the inverse surface area per unit volume, 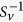, versus time, *t*, see Fig. 2(A). Then, a least-squares fit to the three parameters *t*_0_, *S*_*v*_(*t*_0_), and *K* in Eq. 1 is used to analyze this data. In particular, we fit the 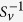 data in the range 1500 ≤ *t* ≤ 5000 s to a line of constant slope (*n* = 1), in red, corresponding to growth in the interface-reaction limit. The regression coefficient is 0.99, indicating that the 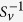 data in this time interval is represented remarkably well with a straight line. Deviations from linear kinetics at late times (i.e., beyond 5000 s) suggest either a change in the supersaturation or a change in the growth mechanism, e.g., from interface-reaction-limited to diffusion-controlled growth. The latter possibility is explored in greater detail in section 0.2.

### Microstructural Characterization

**Figure 3:**
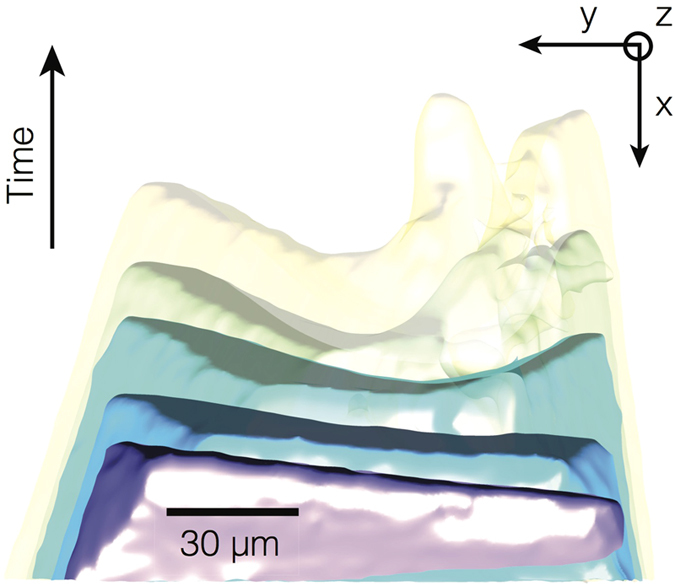
3-D reconstructions superimposed at five time-steps *t* where Δ*t* is 20 s. The Si particle grows from the oxide skin (at bottom, not pictured) into the melt (at top, in white). The initially faceted solid-liquid interface, in purple, develops curvature during growth. Growth accidents may contribute to the proliferation of twin defects and a highly curved interface.

We measure the interfacial dynamics of the same particle on a more local scale. Figure 3 shows the 3-D interfacial morphology at five different times superimposed onto the same image. The time interval between the reconstructions is 20 s. The Si particle grows from the oxide skin (at bottom, not pictured) into the melt (at top, in white). Interestingly, the particle is initially fully faceted, but develops concave (negative) curvature relative to the solid phase during growth. We attribute this morphological instability to the formation of twin defects during growth, as explained further in section 0.3.

**Figure 4:**
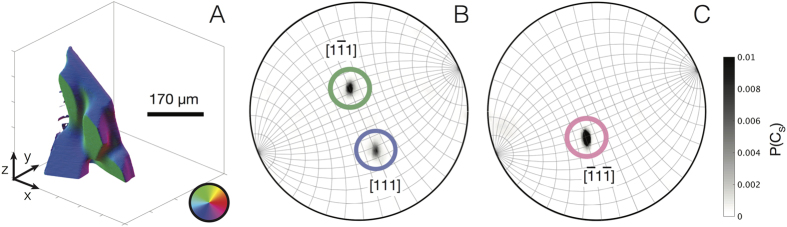
(**A**) The 3-D microstructure of the Si particle illuminated according to the orientation of the surface normals 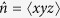. A given 〈*xyz*〉 orientation (in the specimen frame) maps to a unique red-green-blue (RGB) color triplet indicated by the color wheel (see inset). Two stereographic projections are needed to fully quantify the directionality of the Si particle: one along (**B**) 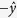, and the other along the (**C**) 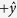 direction. The grid spacing in both measures 10°. The three peaks in the stereographic projections represent the orientations of the three largest facets of the Si particle. The facet circled in pink is nearly 180° from the facet circled in green, i.e., they are on opposite sides of the Si particle. We assign the blue and the green facets in (**A**,**B**) to the {111} family, and assuming that there are no defects (e.g., grain boundaries) between these facets, they must belong to the same monocrystal. As a consistency check, the angle between the blue and green facets is 72°, which matches well with the 70.53° dihedral angle of a monocrystal bounded by {111} habit planes.

In order to further elucidate the structure of the solid-liquid interfaces, we characterize the distribution of interfacial orientations during growth. Figure 4(A) shows the Si particle at an early stage in the growth process, illuminated according to its orientation in the specimen frame. That is, a given surface normal 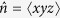 corresponds to a unique red-green-blue (RBG) color triplet, see the inset color wheel in Fig. 4(A). To fully quantify the directionality of this Si particle, we plot two stereographic projections: one has a zone axis of 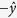 (Fig. 4(B)) and the other has a zone axis of 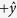 (Fig. 4(C)), where 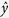 is one of the axes in the specimen coordinate system, *C*_*S*_. The colorbar gives the probability *P* of finding a particular orientation in the stereographic projections. The peaks in Fig. 4(B,C) represent the orientations of the three largest facets (by area) in the Si particle. To aid in cross-checking the real space and projection data, the prominent green facet in Fig. 4(A) is circled in green in Fig. 4(B), and so on. However, from this particular vantage point, the facet circled in pink (Fig. 4(C)) cannot be seen in the 3-D reconstruction as it is on the opposite side of the Si particle, 180° away from the green facet. Thus, the pink and green poles in Fig. 4(B,C) are related by inversion symmetry. In addition, we index the prominent blue and green facets to the {111} family. This is plausible because the kinetic^8^ and equilibrium Wulff shapes^10^ of Si are bounded by {111} planes. As explained in section 0.7.2, provided that these two facets–which are adjacent to one another–have no defects between them, they must belong to the same monocrystal. To verify the above, we calculate the angle between these two facets as 72°, which matches well with the 70.53° dihedral angle of a monocrystal bounded by {111} habit planes. This information can now be used to construct the orientation matrix *g*, such that *all* crystallographic orientations can be measured *relative* to this parent monocrystal, see 0.7.2 for computational details. In what follows, we rotate the specimen coordinates to the crystallographic coordinates of the parent monocrystal identified in Fig. 4.

**Figure 5:**
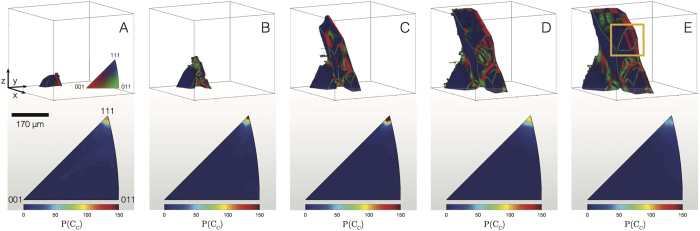
Evolution of the solid-liquid interfacial texture during polycrystalline Si growth: the top row displays the crystal facets of the Si particle illuminated according to the standard triangle in (**A**), and the bottom row depicts the corresponding inverse pole figures (IPFs). Shown are five representative time-steps during growth: (**A**) 930 s (895 °C), (**B**) 1476 s (885 °C), (**C**) 3071 s (859 °C), (**D**) 5254 s (822 °C), and (**E**) 8230 s (773 °C). The limits of the color-bar range are fixed such that multiple IPFs can be compared. From the IPFs, it can be seen that the probability of the {111} orientation relative to the parent crystal, *P*({111}), first increases and then decreases during growth. The boxed region in (**E**) consists of lamellar grain boundaries, which are characterized in Fig. 6.

**Figure 6:**
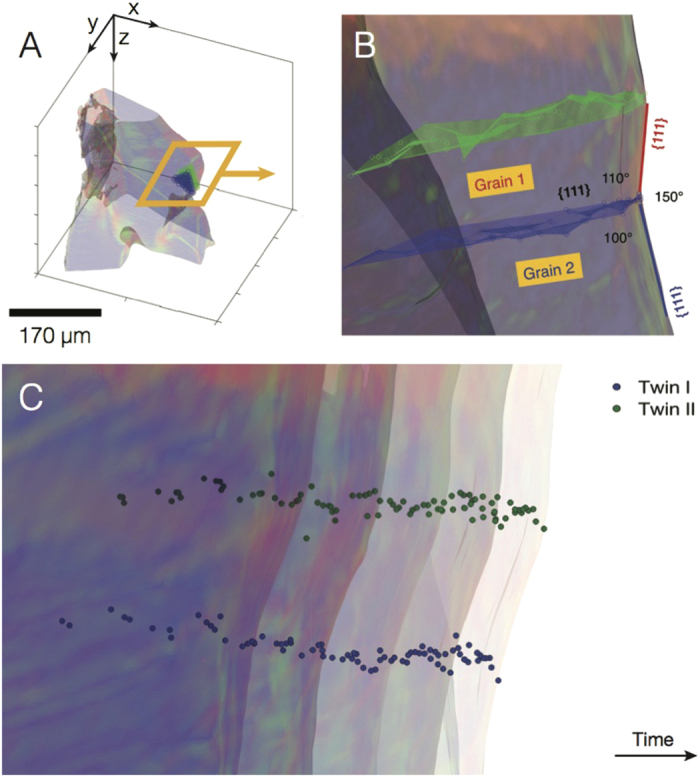
Characterization of lamellar grain boundaries using 4-D XRT data. (**A**) Semi-transparent 3-D microstructure of Si particle overlaid with meshes of the nearly planar grain boundaries (in blue and green). (**B**) Magnified bird's eye view of the boxed region in (**A**). The planarity of the grain boundaries suggests a high degree of coherency between the adjoining grains. The boundaries make an angle of approx. 110° with respect to the facet planes, which corresponds to the angle between two {111} interfaces of the same family. Thus, the lamellar grain boundaries are coherent {111} Σ3 (twin) boundaries. The angle of the re-entrant edge is 150°. (**C**) Propagation of the twinned interface in (**B**) during growth. Five semi-transparent isochrones are plotted (Δ*t* ≈ 600 s) together with points along the two twin boundaries, labelled *I* and *II* and colored in blue and green, respectively. The color of the particle interfaces indicates the passage of time, thereby making the interfacial evolution more clear. The morphology of the twinned interface appears nearly identical between time-steps, i.e., the re-entrant groove does not disappear during growth, consistent with the Wagner-Hamilton-Seidensticker (WHS) model^14^,^15^.

Once the crystallographic normals are calculated for all interfacial patches at all time-steps, we can track the textural development of the solid-liquid interfaces during the growth process. Figure 5 (top row) displays the Si particle at five representative time steps, illuminated according to the standard triangle given in Fig. 5(A). While the broadest facets have the {111} orientation (in navy blue), other crystallographic orientations are certainly evident, indicative of polycrystallinity. Note that the crystallographic orientation is measured *relative* to the parent monocrystal. If the Si particle were to be single crystalline, then all interfaces would be navy blue. On the other hand, surfaces colored other than navy blue indicate {111} interfaces of a twin orientation. That is, the local coordinate system changes upon twinning. For instance, the boxed region in Fig. 5(E) shows multiple grains that meet at lamellar grain boundaries. This microstructure resembles that of parallel annealing twins in face-centered cubic (FCC) metals^26^, a possibility that is explored further in Fig. 6. The bottom row in Fig. 5 shows the five corresponding inverse pole figures (IPFs). The limits of the color-bar range are fixed such that multiple IPFs can be compared. It can be seen that the probability *P* of the {111} orientation first increases and then decreases during the growth process.

Patches of interface are indexed as {111} relative to the parent crystal if the orientation of the patches falls within the yellow region of the IPF, see the inset in Fig. 2(B). The boundary of this yellow region is selected somewhat arbitrarily, but it does not affect the qualitative temporal evolution of the fraction of interfaces with the {111} orientation. Then, the total area fraction, *A*_*A*_, of these {111} patches is plotted over time *t* (Fig. 2(B)). We find that the maximum areal coverage of the {111} orientation relative to the parent monocrystal (identified in Fig. 4) is around 45% and occurs at approx. 3200 s. For *t* < 3200 s, the high mobility facets grow out and cease to exist such that the crystal is asymptotically bounded by the low mobility^7^,^8^ {111} planes. This kinetic behavior might account for the regime of {111} facet growth observed for *t* < 3200 s. On the other hand, the proliferation of defects, e.g., twin boundaries, at later times (*t* > 3200) leads to the formation of new grains that have different orientations from that of the original, {111}-bounded monocrystal. It is important to emphasize that we have plotted only those {111} interfaces in the crystallographic frame of the original, parent crystal; in other words, the {111} orientations of the twinned regions are not accounted for in Fig. 2(B). This might explain the decrease in the areal coverage of the {111} orientation upon twinning. The plot of 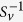 versus *t* (Fig. 2(A)) can now be understood in the context of *A*_*A*_ {111}: when *A*_*A*_ {111} is high, such as in the early stages of growth, 2-D nucleation on {111} is needed to initiate new layers and thus growth is interface-reaction limited. Conversely, it is concievable that growth is governed by the bulk diffusion of solute at late times provided that there are enough defects to facilitate the growth process.

We can examine more closely the local structure of the solid-liquid interfaces (Fig. 5(E)) using our 4-D XRT data, as follows: at each time-step, we measure by hand several points along the crease of the triple line in the interface; then, we fit these data points to 3-D surfaces. Assuming that the crease between the red and blue interfaces in Fig. 5(E) is due to an internal grain boundary (as we will see, a twin boundary), the path of the crease with time informs the location of the grain boundary within the microstructure. Figure 6(A,B) show the semi-transparent microstructure of the Si particle overlaid with the grain boundaries (in green and blue). The mesh surfaces of these boundaries are nearly planar, indicating a high degree of coherency between the adjoining monocrystalline grains. Boundaries are termed incoherent if there exists any lattice curvature that must be accommodated plastically through dislocations, which is not the case here. In Fig. 6(B), the grain boundary in blue makes an angle of approx. 110° with respect to the facet planes, which corresponds very nearly to the 109.47° angle between two {111} interfaces of the same family. Also, the angle of the concave triple junction measures 150°; this is within 10% error of the 141.06° re-entrant groove angle centered at a Σ3 twin plane. From these angular relationships, we are able to classify the lamellar grain boundaries as {111} Σ3 twin boundaries. Furthermore, the grain boundary misorientation measured here *via* 4-D XRT is consistent with that measured using EBSD for the case of coarsened Si particles in a eutectic matrix^25^. Shahani and colleagues^25^ found that multiple {111} Σ3 twin boundaries intersect the “rough” edges of the Si particles.

The propagation of this doubly-twinned interface is depicted in Fig. 6(C). Five semi-transparent isochrones are plotted (Δ*t* ≈ 600 s) together with points along the two twin boundaries, labelled *I* and *II* and colored in blue and green, respectively. The interfacial response is identical between time-steps, i.e, the re-entrant groove does not disappear during growth. This picture is consistent with the WHS model, and incongruous with the F model, as will be discussed in section 0.4. In particular, we do not see the appearance and disappearance of 60° triangular corners expected from the F model.

**Figure 7:**
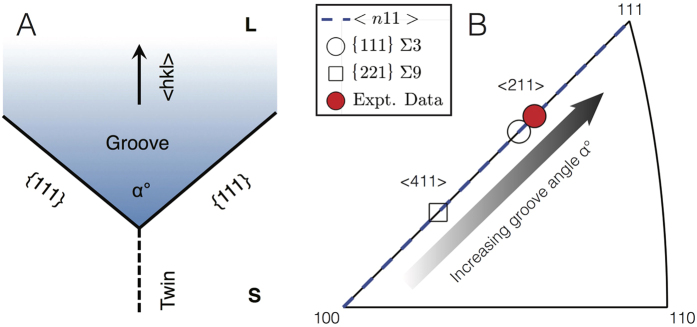
(**A**) Schematic showing the morphology of a twinned interface near a re-entrant groove. The groove has angle *α* and is bounded by two {111} habit planes. The generalized growth direction is denoted 〈*hkl*〉. (**B**) The growth direction of a twinned interface 〈*n*11〉 is plotted on the stereographic triangle, where *n* varies with *α* according to Supplementary Eq. 2, and *α* ∈ (70.53°, 180°]. Also plotted are the growth directions for a {111} Σ3 boundary (*α* = 141.06°), a {221} Σ9 boundary (*α* = 109.47°), and the experimental data (*α* = 150°, see Fig. 6). Thus, the twinned Si particle in our study grows along the 〈211〉 direction.

In general, five macroscopic parameters are needed to classify a grain boundary^6^. Three parameters define the orientation relationship between the two adjacent grains, usually in terms of the Eulerian angles *ϕ*_1_, Φ, and *ϕ*_2_. Two more parameters define the inclination of the grain boundary plane, expressed as the plane normal 〈*uvw*〉. In our work, we determine all five parameters from our tomography data: grain orientations are found from the crystallographic interface normal distribution (CIND) construction (Fig. 5) and the boundary plane orientation from the interfacial isochrones (Fig. 6). All intrinsic properties of the grain boundaries are functions of these parameters^27^. For instance, we demonstrate in the Supplementary Information that the growth direction of the twinned interface is fixed by the geometry of the re-entrant groove. For a groove of angle *α* between two {111} habit planes, as in Fig. 7(A), the growth direction 〈*hkl*〉 is 〈*n*11〉 where *n* is a function of *α* (see Supplementary Eq. 2). This function *n*(*α*) is plotted on the standard triangle as *α* varies from 70.53° to 180° (dashed line). Note that *α* = 180° represents a flat {111} facet growing in the 〈111〉 direction. Also plotted are the growth directions for a {111} Σ3 boundary (*α* = 141.06°), a {221} Σ9 boundary (*α* = 109.47°), and the experimental data (*α* = 150°, see Fig. 6). Thus, the twinned interface in our study grows along the 〈211〉 direction. It has been suggested^28^ that the second-order Σ9 boundary may appear at very high growth rates, but we do not observe any such twinning configuration in our work. In addition, we find no evidence for cozonal twins^28^ with effective 〈110〉 or 〈100〉 fiber axes.

**Figure 8:**
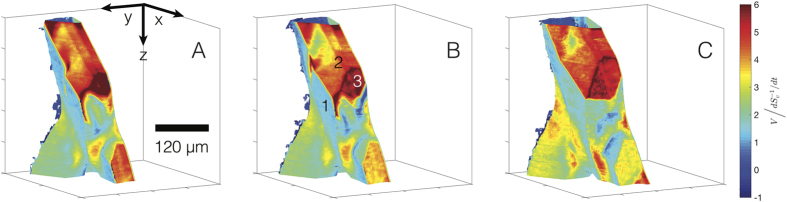
3-D microstructure of the Si particle during the late stages of growth, illuminated according to interfacial velocity *V*. Shown are three representative time-steps: (**A**) 4856 s (829 °C), (**B**) 6047 s (809 °C), and (**C**) 8230 s (773 °C). *V* is scaled by 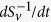 and the color-bar range is fixed such that the three plots can be compared. The numbering in (**B**) indicates features-of-interest: (1) {111} facet plane, (2) ridge structure, and (3) re-entrant groove. It can be seen that, in general, 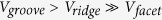. Thus, the re-entrant groove serves as a favorable attachment site for solute atoms during growth.

Twin-mediated growth theories^14^,^15^,^16^,^17^,^18^,^19^,^20^,^21^ predict that re-entrant grooves serve as preferential attachment sites for solute atoms during crystal growth. We confirm this notion by illuminating interfacial patches according to normal velocity *V*, as shown in Fig. 8 at three representative time-steps. *V* is scaled by 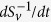 and the color-bar range is fixed such that the three plots can be compared. The numbering in (b) indicates features-of-interest: (1) {111} facet plane, (2) ridge structure, and (3) re-entrant groove. Recall that the ridge structure is convex while the re-entrant groove is concave with respect to the solid Si phase. In general, we detect that 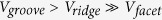, as one would expect based on the bonding arguments presented in the introduction. Thus, we have established unequivocally *via* 4-D XRT the properties of the twinned interface, including its crystallography and dynamics.

## Discussion

### Formation of Twin Defects

It has long been recognized^29^ that, due to the relatively low stresses experienced by the crystal during growth, the formation of twin defects (Fig. 3) cannot be of a mechanical origin and must therefore be related to solidification, and hence to some phenomenon occurring at the solid-liquid interface. There has been much debate on *when* and *where* such growth twins form, i.e., whether the critical nucleus originally contains a twin boundary^30^,^31^ or whether the twin boundaries form after nucleation^32^,^33^,^34^. Our results in Fig. 3 suggest the latter possibility, that multiple twin defects perturb the initially faceted surface of a Si monocrystal during bulk crystal growth. According to the “growth accident” hypothesis^33^, twins are formed and terminated by errors of the stacking of the {111} planes. Cullis and colleagues have observed *via* the pulsed laser melting technique the profuse twin nucleation on close-packed {111} lattice planes; eventually, the multiplication of these growth accidents at the solid-melt interface leads to the formation of an amorphous solid phase^35^,^36^. Moreover, the twinning probability is negatively correlated with the stacking fault energy (SFE). For instance, Σ3 {111} boundaries possess very low SFE (30 mJ/m^2^)^37^,^38^, less than one-fifth of the SFE of other grain boundaries, and hence appear most frequently in grain boundary character distributions^38^. Fujiwara and colleagues^32^ proposed a mechanism by which growth accidents on Si {111} lead to the formation of parallel Σ3 {111} boundaries. While we observe lamellar twins in our work (see Fig. 6), the formation of such defects involves sub-micrometer scale dynamics that are well below the spatial resolution of our current XRT experimental results. Future improvement of higher resolution dynamic XRT would provide more conclusive evidence.

It should be noted that crystal twinning is also sensitive to the chemical environment. According to the “impurity induced twinning” (IIT) mechanism proposed by Lu and Hellawell^39^, the adsorption of impurity atoms at monolayer steps may contribute to an alteration of the stacking sequence of {111} planes, and thus to the formation of twins. Geometrical considerations^39^ require a specific radius ratio of impurity to Si for IIT to take place. However, neither Al nor Cu in the Al-Si-Cu alloys of this study satisfies this condition. Instead, Timpel and colleagues^40^ suggest that it is not the geometrical *size* factor that plays a major role in IIT but rather the *chemistry* of the co-segregations. Thus, we cannot rule out the possibility of intermetallic Al-Si-Cu adsorbents.

### Growth of Twinned Polycrystal

The morphology of a twinned crystal results from a complex interplay between nucleation at the re-entrant groove and lateral growth of the layers so initiated. Let the nucleation rate be denoted by 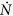 and lateral growth by *V*_*l*_. Consider three regimes: (i) 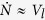, (ii) 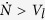, and (iii) 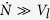. In case (i), the lateral growth of layers away from the groove can keep pace with the nucleation rate at the re-entrant; as such, the corners of the crystal remain large, with wide terraces and flat interfaces. This “birth-and-spread” of new layers, emanating from the type *I* re-entrant groove, is the model most frequently encountered in the literature^14^,^15^. If instead 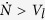 (case (ii)), many layers emerge from the re-entrant. The lateral growth cannot keep up with the nucleation rate and the interface resembles a multiply tiered “wedding cake” centered at the twin boundary. By increasing 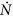 further relative to *V*_*l*_ (case (iii)), the re-entrant accumulates a multitude of layers that are stacked one above the other. At the macroscale, this configuration may have the appearance of a triangular mound projecting into the melt. Clearly, then, a continuum of polycrystalline growth forms can be expected based on the kinetics of the materials system investigated.

Our results (Figs 5 and 6) show that the type *I* re-entrant groove does not disappear during growth, suggesting that the lateral growth of layers, *V*_*l*_, keeps pace with the nucleation rate, 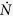, i.e., 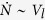. This is consistent with the WHS picture of twin-mediated crystal growth^14^,^15^. On the other hand, the F model predicts the formation of triangular 60° corners at the type *I* groove^20^,^21^. Differences between the F and WHS approaches can be rationalized based on nucleation rates at the type *I* re-entrant, noting that, in general, 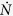 depends strongly on temperature, undercooling, and the associated energy barrier^41^. While the nucleation barrier is not well known, and may itself be a function of alloy composition, it is worth mentioning that the experiments performed by Fujiwara *et al*.^20^,^21^,^22^,^23^ were conducted at temperatures much higher than in this investigation. In particular, Fujiwara *et al*. studied the crystallization of pure Si (melting temperature of 1414 °C) while we observed the growth of Si in an Al-Si-Cu melt (liquidus temperature of 910 °C). If we consider the influence of absolute temperature on 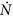 alone, we might expect 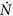 to be considerably larger in the experiments performed by Fujiwara *et al*., resulting in the pancake stacking of layers at the type *I* groove (cases (ii) or (iii) above). Thus, a single growth mechanism may be inadequate to explain interfacial phenomena over the entire (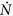, *V*_*l*_) parameter space. Instead, the F model may be operant in the limit of large 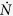 while the classical WHS model may be more appropriate in the limit of small to moderate 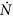 (relative to *V*_*l*_).

## Conclusion

We have for the first time provided direct, experimental observations of the classical WHS mechanism of twin-mediated crystal growth^14^,^15^, proposed over 50 years ago. Our time-resolved data collected *via* 4-D XRT has made it possible to track with high precision the interfacial dynamics of polycrystalline Si particles in an Al-Si-Cu liquid. In particular, we have quantified the local velocities and crystallographic orientations of the solid-liquid interfaces during growth. Our results show, unambiguously, that the type *I* re-entrant groove does not disappear during the continuous propagation of a doubly twinned interface, consistent with the WHS mechanism. Such growth morphologies are attainable when the nucleation rate at the type *I* re-entrant groove is comparable to the lateral growth of layers so initiated. The novel experiment and analysis method developed in this work provide a unique vision into the growth behavior of polycrystalline materials. Along with the temporal and spatial resolution improvements in 4-D XRT, this method will facilitate the precise dynamic observation of crystal growth and improve our understanding of the growth forms that arise during materials processing.

## Methods

### Experimental Methodology

Alloy buttons of composition Al-32wt%Si-15wt%Cu were prepared *via* vacuum arc-melting at the Materials Preparation Center (MPC) at Ames Laboratory, using 99.999% purity Al, 99.9999% purity Si, and 99.99% purity Cu. The powders were repeatedly melted on a water-cooled Cu hearth plate in an atmosphere of high purity Ar. Each button was flipped three times to ensure homogeneity. Then, the buttons were machined into rods measuring 1.1 mm in diameter by 5.5 mm in length, using electrical discharge machining (EDM) at Northwestern University. To remove any impurity atoms that may be adsorbed onto the surfaces of the sample, the rod surfaces were etched with a 1:1 solution by volume of 70% HNO_3_ and de-ionized water using a cotton swab for five minutes. The alloy compositions were confirmed quantitatively *via* energy dispersive X-ray spectroscopy (EDS) prior to the tomographic investigation.

At temperatures relevant to the study of primary Si growth, the solid solubility of Cu in Si is less than 0.5 ppm^42^,^43^; in other words, the Cu constituent is entirely dissolved in the liquid phase. Thus, the heavy element Cu acts as a contrast agent between the liquid and solid phases, thereby enabling the visualization of the Si particles in the conventional attenuation mode. This has been demonstrated previously by Mathiesen *et al*.^44^ and Shahani and colleagues^45^. Attenuation contrast XRT experiments were carried out at sector 2-BM at the Advanced Photon Source (APS) at Argonne National Laboratory (ANL). The high flux “pink” X-ray beam (10^14^ photons/s/mm^2^) was focused on the samples and a 20 *μ*m thick LuAg:Ce scintillator was used to convert the transmitted X-rays to visible light. High resolution imaging was performed utilizing a PCO Edge CMOS camera equipped with a 10x magnifying objective to provide pixel sizes of 0.65^2^ *μ*m^2^. The X-ray field-of-view measured 1664 *μ*m in width by 715 *μ*m in height.

During the experiment, the samples were heated in a resistive furnace to above the liquidus and allowed to equilibrate at 910 °C. The molten specimens were held by their own oxide skin. Then, the samples were cooled at a rate of 1 °C/min while projections were recorded. Given the 1.1 mm diameter of the sample, the temperature distribution was assumed to be uniform within the sample. For the first 20 minutes, projections were collected continuously at a rate of 50 frames/s, with 1500 frames distributed evenly in every 180° rotation; these parameters provide a temporal resolution of 30 s between each of the 40 reconstructions. For the next 120 minutes, 40 tomographic scans with the same parameters were spaced 150 s apart. The motivation for this data collection scheme is that the growth rate of the particle is inversely proportional to its system-average length-scale in the bulk diffusion controlled limit^46^; as such, one scan every three minutes during the late stages of growth is adequate to keep up with the interfacial dynamics. In summary, we collected a total of 80 tomographic scans during particle growth, producing over 670 GB of data.

### Data Processing Pipeline

Following the synchrotron experiment, the tomographic data were processed off-site using the Python-based toolbox TomoPy^47^. Within the TomoPy framework, we (i) normalized the data with respect to the dark-field (offset) and white-field (gain) projections; (ii) estimated the center-of-rotation of the rod sample *via* entropy-based optimization^48^; (iii) corrected for stripe artifacts in the sinogram domain *via* combined wavelet mbox-Fourier filtering^49^; and (iv) completed tomographic reconstruction using the Gridrec algorithm^50^, see the work by Gursoy *et al*. and references therein^47^. These processing steps were performed on a Mac Pro 3.5 GHz, 12-core Intel Xeon system with 64 GB RAM.

The remainder of the data processing and microstructural analyses was based on routines written in MATLAB R2015b^51^. Specifically, we applied a combination of median filtering and morphological operations^52^ on each 2-D reconstruction image (along the axis of rotation), in order to minimize the speckle noise caused by Nyquist undersampling. The filtered 2-D images were segmented with an appropriate threshold, and combined to reveal the 3-D microstructures. For the subsequent analysis, we focused on the growth dynamics of a single particle within the tomographic field-of-view. This represents a subset of the 3-D volume, measuring 465 × 582 × 325 *μ*m^3^. The digitized surfaces of this particle were meshed; to remove any staircasing artifacts, we smoothed the triangular mesh by mean curvature flow^53^. Each triangle in the mesh is referred to as a patch of interface. The following calculations involving (i) surface area per unit volume, (ii) crystallographic normal distribution, and (iii) interfacial velocity make use of mesh face and vertex positions.

### Microstructural Analyses

#### Surface Area Per Unit Volume

The growth rate of the Si particle may be limited by either (i) the diffusion of growth species from the bulk to the growth surface, or (ii) the adsorption of the growth species onto the growth surface. Consider an isolated spherical particle in a supersaturated matrix: if the growth process is controlled by diffusion, then the particle radius varies parabolically with time; on the other hand, the particle radius varies linearly with time for interface-reaction-limited growth^46^. In other words, the growth mechanism can be identified by tracking the particle radius over time. Since the Si particle is highly anisotropic, we seek another time-dependent length-scale to characterize the system. One such possibility, suggested by Marsh and Glicksman^54^, is the inverse solid-liquid surface area per unit volume, 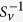. The subscript *v* is the volume of the particle. Thus, assuming growth follows a power law relationship, 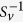 varies as


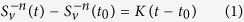


where *S*_*v*_(*t*_0_) is the specific surface area at time *t*_0_, representing the onset of steady-state growth; *K* is a rate constant that depends on materials parameters; and *n* = 1 in the interface-reaction limit or *n* = 2 in the diffusion limit. To adequately represent the kinetics of particle growth, we measure *S*_*v*_ at each time-step according to


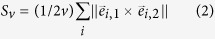


where 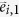 and 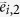 are edge vectors of mesh triangle *i*, × indicates cross product and ||∗|| is the vector norm.

#### Crystallographic Normal Distribution

While being morphology-independent, the length scale 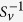 is an average over the system and cannot account for any localized evolution of the microstructure. In order to measure these microstructural details, we characterize the distribution of crystallographic orientations of the solid-liquid interface during growth. We begin by calculating the unit normal vectors along the solid-liquid interface, in which all normals point from the Si particle to the liquid, following thermodynamic convention. The interface normal 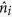 of a mesh triangle *i* with edge vectors 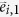 and 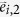 is defined as


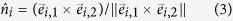


The normals 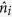 are measured in the specimen coordinate system, which is relative to the tomographic detector plane; in the specimen frame, the 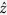 direction is parallel to the axis of rotation of the sample. To rotate the specimen coordinates *C*_*S*_ onto the crystal coordinates *C*_*C*_ we specify an orientation matrix *g* such that *C*_*C*_ = *g* *C*_*S*_^6^. The 3 × 3 orientation matrix *g* describes the three rotations by the Eulerian angles *ϕ*_1_, Φ, and *ϕ*_2_, necessary to bring *C*_*S*_ into coincidence with *C*_*C*_. Thus, the interface normal of triangle *i* in the crystallographic frame is 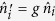. The point group *m*3*m* of the diamond structure has 48 symmetry elements; so, to generate all of the crystallographically-related solutions we premultiply the orientation matrix *g* by each of the 48 3 × 3 symmetry operators *T*_*j*_^6^,^55^. Thus, the crystallographic orientation of the solid-liquid interface is given by


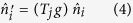


Once 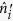 is calculated for all mesh triangles, the distribution of 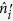 is plotted as a stereographic projection (inverse pole figure) along 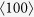, hereby referred to as a crystallographic interface normal distribution (CIND). According to crystal symmetry it is not necessary to show the entire distribution, but one unit triangle suffices. In this way, each orientation from a sample population is represented only once^6^. This well-known unit triangle or “fundamental zone” is bounded by 〈100〉, 〈110〉, and 〈111〉. For all time-steps the standard triangle can be plotted, thereby providing quantitative snapshots of the textural evolution during the growth process. Our method of characterizing crystallographic orientation is similar to that first popularized by Rohrer and colleagues^56^,^57^,^58^, except these authors look at the *internal* boundaries in a material, while we consider only the external, solid-liquid interfaces visible in our tomographic data. Rowenhorst^59^ uses a similar approach in his CIND construction, but does not account for crystallographic symmetries.

The orientation matrix *g* remains to be determined in order to apply Eq. 4 to the experimental data. The analysis of facet crystallography (and hence *g*) can be performed *ex situ via* electron back-scattered diffraction (EBSD)^6^, but we describe below that *g* can be easily determined using our tomography data. Our analysis relies on prior knowledge of the structure of Si polycrystals: namely, that the kinetic^8^ and equilibrium Wulff shapes^10^ are dominated by {111} planes. Thus, the broad, flat facets of the Si particle of the parent crystal can be indexed as {111} without any loss of generality. Provided that there are no defects between two adjacent facet planes, they must belong to the same monocrystal. As a consistency check, the dihedral angle between two {111} facet planes in a monocrystal must equal 109.47°. Once the facet planes have been confirmed as {111} orientations, the optimal rotation matrix *g* is calculated using the Kabsch algorithm^60^, which is based on singular value decomposition (SVD). Shechtman^61^,^62^ used an analagous procedure to identify facet planes in diamond films produced by chemical vapor deposition (CVD). His analysis requires only standard scanning electron microscopy (SEM) and is based upon an understanding of the nature of diamond twins.

#### Interfacial Velocity

The collection of *in situ* 4-D tomography data enables further analysis of the Si particles during growth. In particular, we can calculate the interfacial velocity 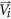 of all triangles *i* in the mesh, where 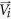 is measured in a direction normal to the interface. Conventionally, this has been accomplished using the ray-triangle intersection algorithm proposed by Möller and Trumbore (hereby denoted MT)^63^. Recently, however, Shahani and colleagues^45^ demonstrated that interfacial velocity can be calculated using the k-nearest neighbors (k-NN) algorithm^64^ which offers substantial speedup compared to the MT method. Therefore, we use the k-NN algorithm to compute 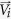 at all time-steps, see the work by Shahani *et al*.^45^ for computational details.

## Additional Information

**How to cite this article**: Shahani, A. J. *et al*. Twin-mediated crystal growth: an enigma resolved. *Sci. Rep.* **6**, 28651; doi: 10.1038/srep28651 (2016).

## Supplementary Material

Supplementary Information
