# Supplementary material for: Twin-mediated crystal growth: an enigma resolved
Source: Sci Rep. 2016 Jun 27;6:28651. doi: 10.1038/srep28651 (PMC4922011; doi:10.1038/srep28651)
Supplement: Supplementary Information [file srep28651-s1.pdf]

Twin-mediated crystal growth: an enigma resolved

Ashwin J. Shahani[1,*], E. Begum Gulsoy[1], Stefan O. Poulsen[1], Xianghui Xiao[2], and Peter W. Voorhees[1,*]

[1]Department of Materials Science and Engineering, Northwestern University, Evanston, IL 60208, USA
[2]Advanced Photon Source, Argonne National Laboratory, Lemont, IL 60439, USA
*shahani@u.northwestern.edu, p-voorhees@northwestern.edu

Supplementary Information

Growth Mechanisms of Twinned Interface

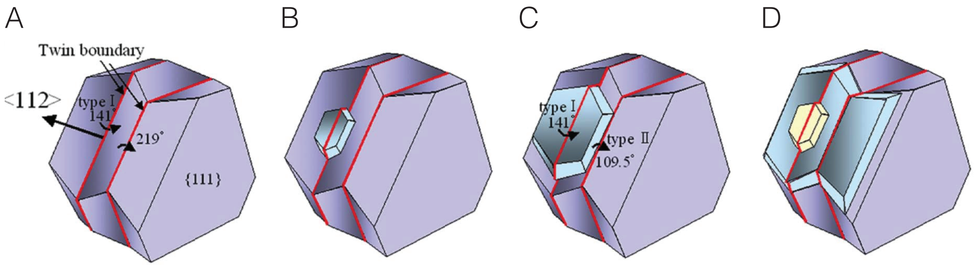

Supplementary Figure 1. Idealized schematic of growth process of (a) crystal with two parallel twin planes (in red), according to Wagner, Hamilton and Seidensticker.[14,15] (b) Nucleation first occurs at the 141.06° re-entrant corner (type *I*). (c) The initiated layer propagates to the second twin plane and forms a new corner measuring 109.47° (type *II*). (d) Nucleation then occurs at the type *II* corner, leading to the continuous propagation of the crystal in $\langle 112 \rangle$ direction. Reproduced with permission from Ref. [20]

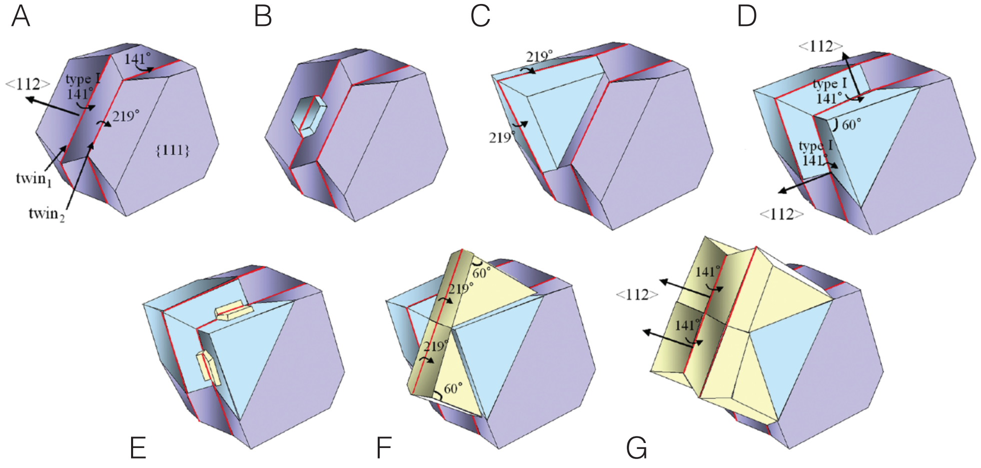

Supplementary Figure 2. Growth process of (a) crystal with two parallel twin planes (in red), according to Fujiwara and colleagues.[20] (b) Similar to Supplementary Fig. (b), nucleation first occurs at the type *I* re-entrant corner, where $twin_1$ intersects the crystal surface. (c) A triangular corner measuring 60° at the tip is formed due to the rapid growth at the groove. (d) The triangular corner propagates across $twin_2$, leading to the formation of two new type *I* grooves centered on $twin_2$. (e-g) Rapid growth occurs at the new type *I* corners, thereby regenerating the type *I* corners at $twin_1$. This process from (a) to (g) occurs continuously such that the net growth direction is $\langle 112 \rangle$. Reproduced with permission from Ref. [20]

Weight Fraction of Silicon Particles

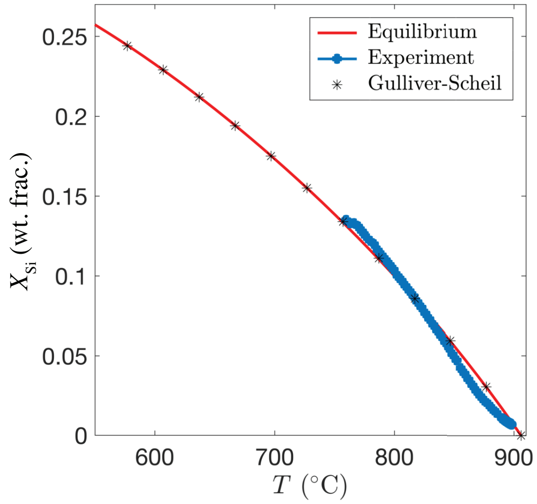

Supplementary Figure 3. Weight fraction of primary Si, X_{Si} versus temperature, T, during growth in an alloy of composition Al-32wt%Si-15%Cu. X_{Si} is calculated from the experimental data over the entire field-of-view and is shown in blue, while equilibrium and Gulliver-Scheil simulations are determined from CALPHAD and plotted as a red line and black stars, respectively. All three curves are nearly coincident, indicating that the growth rate of the solid can keep up with the quench rate. Errors in the measurement of X_{Si} from the experimental data due to small segmentation errors are negligible.

Growth Direction of Twinned Interface

The growth direction of the twinned interface is fixed by the geometry of the re-entrant groove, see Fig. 7(a). Denote the growth direction as $\langle hkl \rangle$, and re-entrant angle as α. The facet planes on either side of the groove have the $\{111\}$ orientation. The growth direction $\langle hkl \rangle$ must satisfy the following two geometric constraints:

$$\begin{aligned} \langle hkl \rangle \cdot \langle 0\bar{1}1 \rangle &= 0 \\ \langle hkl \rangle \cdot \langle 111 \rangle &= \sqrt{3}\cos(90 - \alpha/2) \end{aligned} \tag{S1}$$

where $\vec{a} \cdot \vec{b}$ indicates the dot product between vectors $\vec{a}$ and $\vec{b}$. According to the first equation, $\langle hkl \rangle$ must lie normal to the zone or symmetry axis ($\langle 0\bar{1}1 \rangle$) between the two $\{111\}$ facet planes; the second indicates that $\langle hkl \rangle$ must make an angle of $90 - \alpha/2$ degrees with the $\{111\}$ plane. Eqs. S1 assume that $\langle hkl \rangle$ is normalized. Solving the simultaneous equations gives $\langle hkl \rangle = \langle n11 \rangle$ where

$$n = \frac{4 + 3\sqrt{2}\sin\alpha}{1 - 3\cos\alpha} \tag{S2}$$

Thus, for a re-entrant angle of 141.06°, caused by the intersection of a $\{111\}$ $\Sigma3$ boundary with the surface, the growth direction is $\langle 211 \rangle$. Note that growth direction satisfies the Weiss zone law, since $\langle hkl \rangle$ lies in the plane of the twin boundary (i.e., $\langle 211 \rangle \cdot \langle \bar{1}11 \rangle = 0$). Provided that α is known, the growth direction can be determined *via* Eq. S2 for any interfacial configuration.
